# Effects of Low-Level Laser Therapy on Orthodontic Tooth Movement: Evaluation of Bony Changes via 3DCBCT

**DOI:** 10.3390/children10020384

**Published:** 2023-02-15

**Authors:** Mohammad Khursheed Alam

**Affiliations:** 1 Orthodontic Division, Preventive Dentistry Department, Orthodontic Division, College of Dentistry, Jouf University, Sakaka 72345, Saudi Arabia; mkalam@ju.edu.sa or dralam@gmail.com; 2 Department of Dental Research Cell, Saveetha Dental College and Hospitals, Saveetha Institute of Medical and Technical Sciences, Chennai 600077, India; 3 Department of Public Health, Faculty of Allied Health Sciences, Daffodil lnternational University, Dhaka 1216, Bangladesh

**Keywords:** low-level laser therapy, photobiomodulation, radicular width, bony changes, CBCT

## Abstract

Objective: The prime objective of this research was to study the effect of low-level laser therapy (LLLT) with an evaluation of bony changes via pre- and post-treatment 3DCBCT in orthodontic malocclusion cases treated with fixed orthodontic appliances. Materials and Methods: Subjects who attended the Orthodontic Clinic, were diagnosed with orthodontic malocclusion, treated with fixed orthodontic appliances, and had pre- and post-management CBCT were included in the study. Patients aged 14 to 25 years who met the inclusion criteria were assigned to two groups, group A (LLLT) and group B (non-LLLT). Group A participants were treated with LLLT therapy as per standard protocol after explaining the nature of the treatment. Group B (non-LLLT) participants were not treated with LLLT therapy and therefore served as the control. LLLT was used in the experimental group after placing each archwire. Interradicular bony changes at depth levels of 1 to 4 (2, 5, 8, and 11 mm) using 3DCBCT were measured as outcome parameters. Results: The information collected was analyzed using SPSS computer software. Mostly insignificant differences were noted among groups for the different parameters (*p* < 0.05). Student’s t-tests and paired t-tests were used to investigate the differences. Experimental Hypothesis: There will be significant differences in the interradicular width (IRW) measurements between the LLLT and non-LLLT groups. Conclusions: The hypothesis was rejected. Upon investigation of prospective changes, most of the measured parameters showed insignificant differences.

## 1. Introduction

Orthodontic patients are mostly concerned with improving their dentofacial esthetics as fast as possible, and having oral health benefits is a secondary concern [1]. The reported average duration of treatment with fixed orthodontic treatment (FOT) ranges between two and three years [2]. However, patients usually expect a maximum treatment duration of a year and a half. In addition, lengthy treatment duration may negatively affect national healthcare system efficiency and private practice efficiency as well. Thus, a shorter treatment duration through the acceleration of tooth movement has long been a subject of concern for orthodontists and patients alike [3].

Orthodontic tooth movement (OTM) is a response of the tooth to external mechanical force that initiates complex cellular interactions leading to the remodeling of bone. To accelerate tooth movement, orthodontists have tried various approaches. Low-level laser therapy (LLLT), also called biostimulation or photobiomodulation, involves applying low levels of red light or near-infrared light to treat different illnesses. It can also be called “low-level laser” or “cold laser” because it uses a lower density of light energy that does not increase the temperature of tissues by more than 1 degree Celsius, unlike other types of lasers which are applied for ablation, cutting, or coagulation of local tissues with heat [3]. In the medical field, LLLT as a modality is considered non-invasive, and it is promising due to the lack of reported side effects [3]. The potential of incorporating LLLT routinely in orthodontic practices without causing any disturbance to patients’ regular treatment schedules to accelerate the OTM and reduce the treatment duration is also promising [4].

Settings of LLLT (100 mW, 7.5 J/cm^2^, total 75 J/tooth) were used previously [4,5,6] and yielded promising results in orthodontic patients in terms of pain perception and root resorption [6] investigated in a Saudi population [5] and tooth movement in a Pakistani population [4]. LLLT’s effects on bony changes, assessed via CBCT acquisition before and after orthodontic treatment, have not yet been investigated. Cone beam computed tomography (CBCT) use has been incorporated in dental offices because of its lower cost and size [7]. Furthermore, modern software can create a 3D reconstruction of the area, which further helps the clinician visualize the area of interest. In current studies conducted on LLLT for OTM, the laser was applied either on a daily basis or there were shorter intervals in between two applications. 

The main objective of this study was to investigate the effect of LLLT on bony changes (interradicular width, IRW) with an evaluation via 3DCBCT in orthodontic cases. More specifically, we sought to compare LLLT and non-LLLT groups concerning IRW bony changes in orthodontic cases via 3DCBCT. 

## 2. Materials and Methods

This was a prospective study on Saudi subjects treated for orthodontic malocclusions angle class I, II, or III, or malocclusions with ectopic canine requiring FOT. Subjects were enrolled at the orthodontic specialist clinic. The pre- and post-treatment CBCT data were gathered from the Radiology Archive, College of Dentistry, Jouf University. 

The sample size was determined through the use of power and sample size calculation software (version 3.1.2). We investigated the effect of LLLT on interradicular bone changes. We planned to study the continuous response variable from independent control and experimental subjects with 1 control per experimental subject. In a previous study, the response within each subject group was normally distributed with a standard deviation of 2.79 [8]. If the true difference between the means of the control and the experimental groups is 5.2, we must include 16 control and 16 experimental participants to be able to reject the null hypothesis that the population means of the control and the experimental groups are equal with a probability (power) of 80%. The type I error probability associated with this test of the null hypothesis is 0.05.

Inclusion criteria:

- A minimum treatment age of 14 years in females and 17 years in males to minimize the effect of residual growth.
- Patients without previous orthodontic treatment history.
- Patients with all permanent teeth erupted (except third molars).
- High-quality records (pre- and post-treatment CBCT acquisitions).

Exclusion criteria:

- Interproximal restorations or caries affecting the dimensions of the dentition and arches.
- Supernumerary or missing dentition.
- Abnormal dentition morphology or size.
- Dentition wear affecting the dimensions of the dentition.
- Medications altering the bone metabolism or tooth movement, e.g., bisphosphonates, corticosteroids, NSAIDs, etc.
- Medical problems, e.g., craniofacial malformation, periodontally compromised dentition, impacted teeth except for the third molars, multiple missing teeth, parafunctional habits, or TMJ dysfunction.

**Figure 1:**
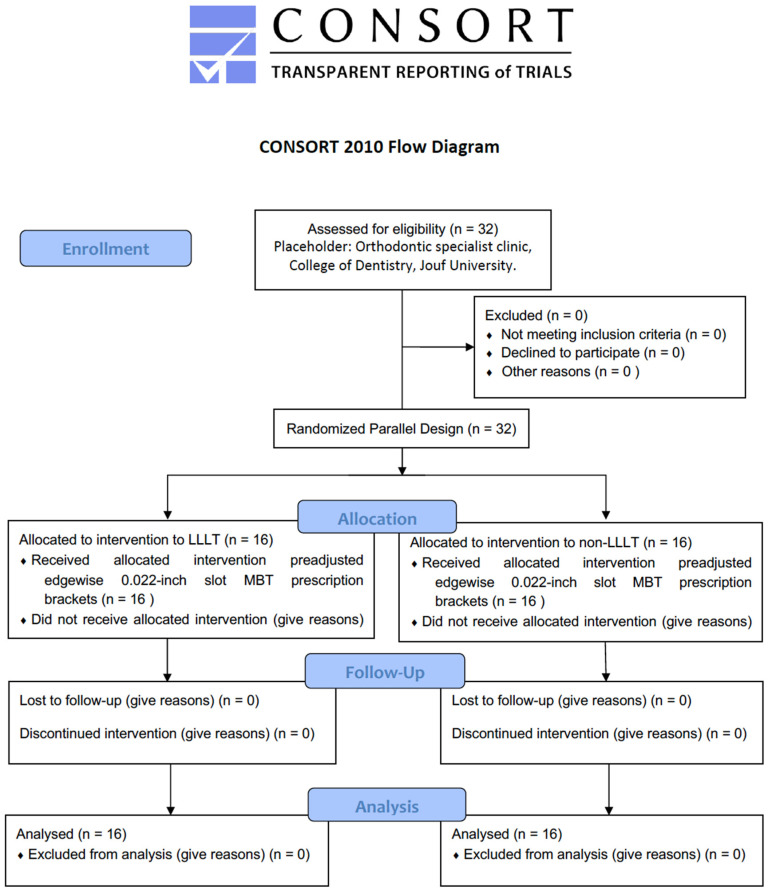
Subject allocation.

Following these inclusion and exclusion criteria, the subjects were randomly allocated into two groups (Figure 1).

Laser Emission/Photobiomodulation: The LLLT unit was a diode laser (iLase; Biolase, Irvine, CA, USA) with power of 100 mW and 940 nm aluminum–gallium–arsenide (Al-Ga-As) set on continuous mode. The optical fiber tip diameter was 0.04 cm^2^. Energy density of 7.5 J/cm^2^ was calculated for each point and 75 J per tooth was the total energy. LLLT was applied to 5 points labially/buccally and palatally on gingival mucosa for 3 s on each point per tooth, starting from the central incisor (#11 and #12) to the first molar (#16 and #26) during each visit. These 5 points were mesial and distal over the cervical third of the root and the middle of the root, and mesial and distal over the apical third of the root. The fiber tip of the laser was held perpendicular to the mucosa covering the tooth roots while in close but light contact with the gingival tissues.

**Figure 2:**
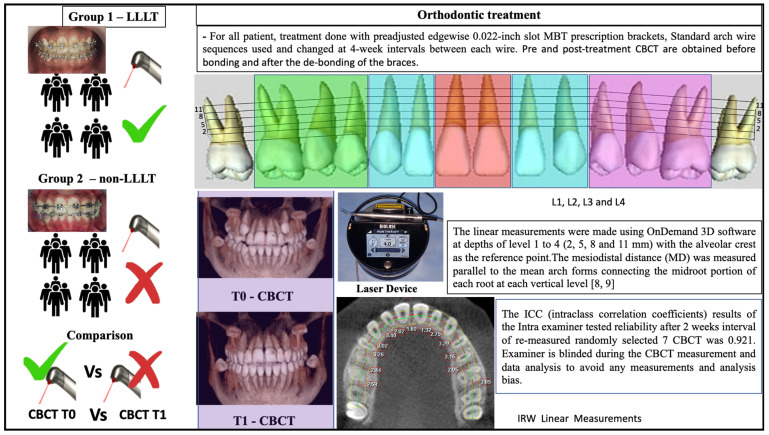
Details of LLLT and IRW linear measurements.

The details of the methodology for the application of the LLLT and the measurements and reliability of the IRW are clarified in Figure 2 [8,9]. CBCT images were acquired before treatment (T0) and immediately after treatment (T1) and were used to measure the IRW changes. The FOT average duration in both groups was 20.015 months, being 19.40 and 20.63 months in the LLLT and non-LLLT groups, respectively. 

### Statistical Analysis

To test and compare the study groups, the paired t-test and independent t-test were used. The analysis was performed using SPSS version 26 (Chicago, IL, USA).

## 3. Results

Pre- and post-treatment (T0 and T1) mesiodistal diameters of the IRW at four different levels (levels 1–4, 2, 5, 8, and 11 mm) were analyzed between two different treatment modalities (LLLT and non-LLLT) in the maxilla. The results are presented by quadrant.

**Figure 3:**
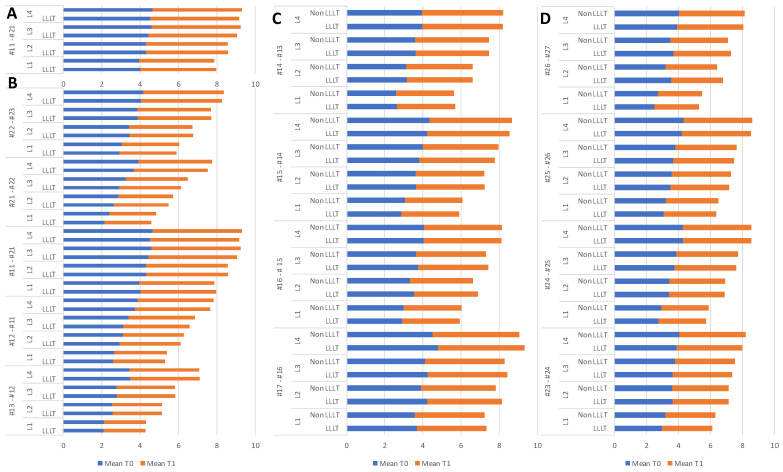
Graphical mean value presentation of IRW of the maxilla (both sides) at various levels. LLLT and non-LLLT differences among pre- and post-treatment of all groups: (**A**) middle quadrant, (**B**) anterior quadrant, (**C**) posterior right quadrant, and (**D**) posterior left quadrant teeth.

Middle quadrant, LLLT and non-LLLT differences: The IRW of the T0, T1, and T0 vs. T1 CBCT of 2 TM (laser vs. non-laser) at the different levels are displayed in Table 1 and Figure 3A. At T0, T1, and T0 vs. T1 CBCT, the data of all four levels are insignificant.

Anterior quadrant, LLLT and non-LLLT differences: The IRW of the T0, T1, and T0 vs. T1 CBCT of 2 TM (laser vs. non-laser) at the different levels are displayed in Table 2 and Figure 3B. At T0, T1, and T0 vs. T1 CBCT, the data of all four levels are insignificant. In general, most of the data showed improvement in T1 compared to T0.

Posterior right quadrant, LLLT and non-LLLT differences: The IRW of the T0, T1, and T0 vs. T1 CBCT of 2 TM (laser vs. non-laser) at the different levels are displayed in Table 3 and Figure 3C. At T0 and T1 CBCT, the data of all four levels are insignificant. T0 vs. T1 LLLT and non-LLLT IRW between #14 and #13 at L1, L2, and L4 showed significant differences.

Posterior left quadrant, LLLT and non-LLLT differences: The IRW of the T0, T1, and T0 vs. T1 CBCT of 2 TM (laser vs. non-laser) at the different levels are displayed in Table 4 and Figure 3D. At T1 CBCT, the data of all four levels are insignificant. T0 vs. T1 laser IRW between #23 and #24 at L4 showed significant differences.

## 4. Discussion

The number of adults seeking orthodontic treatment has been on the rise, but the prolonged duration and associated discomfort of some treatment options are major deterrents. A few techniques have been introduced to accelerate the pace of tooth movement. However, most of the techniques are considered invasive or have reported complications. Therefore, it is necessary to inspect various modalities to overcome these issues for the benefit of patients. The use of LLLT is not only promising for orthodontic treatment but is also used noninvasively in humans for various purposes, without any reported adverse effects [3]. However, most of the lasers being used in medicine and dentistry are classified as type 4 according to the International Electrotechnical Commission (IEC), having the potential to be hazardous especially to the eyes and skin [10]. Therefore, it is necessary to use all protective measures. The advantages of embracing LLLT routinely in orthodontic treatment may enhance the pace of tooth movement without patient discomfort and without disturbing the patient’s routine recall visits. The benefits of using LLLT in terms of changes in the bone after FOT have not been explored. Thus, the present study analyzed the overall outcome after FOT.

The use of CBCT in the dental setting has been on the rise in recent years [11]. CBCT data added valuable three-dimensional insight into the diagnosis and treatment planning process of the dentition and jaws [12]. To investigate outcomes of IRW bony changes after using LLLT, this research used 3DCBCT images. Many previous studies have used CBCT data to produce reliable 3D details of tooth-surrounding tissues and structures after OTM [13,14]. Purmal et al. (2013) [8] and Poggio et al. (2006) [15] also used CBCT data for measuring IRW at different levels. Another study by Bittencourt et al. (2011) [16] used computed tomography (CT) for the IRW measurements. However, CBCT images are superior to CT for the purpose of measuring bony changes. The reason is that CBCT has lower radiation exposure as well as a lower cost to the patient [17]. Currently, there are no published articles describing a similar type of study in a Saudi population. 

At pre- (T0) and post-treatment (T1) CBCT, IRW measurements of #14–#13 were 2.653, 3.161, 3.616, and 3.981 mm at T0 and 3.044, 3.449, 3.855, and 4.215 mm at T1 in the LLLT group. In the non-LLLT group, IRW measurements were 2.595, 3.144, 3.588, and 3.959 mm at T0 and 3.039, 3.454, 3.881, and 4.248 mm at T1. Regarding T0 vs. T1 LLLT and non-LLT groups, IRW measurements between #14 and #13 at L1, L2, and L4 showed significant differences. Purmal et al. (2013) [8] found that at T0, IRW measurements between #14 and #13 at L1, L2, L3, and L4 were 3.01, 3.43, 3.85, and 4.24 mm, respectively; these data are similar to the measured T1 data of the current study. In another study, Poggio et al. (2006) [15] reported IRW measurements at T0 of 3.0, 3.4, 3.9, and 4.3 mm. Bittencourt et al. (2011) [16] found IRW measurements at T0 of 1.6, 1.7, 2.1, and 2.7 mm at four different levels. A point to consider is that significant differences were reported in the morphology of teeth in Caucasians when compared to Asians [18]. This may affect tooth movement and/or the amount of available interdental bone for the use of orthodontic mini-screws or surgical fixation screws. Racial discrepancies have also been reported in the shape and dimensions of the dentition crowns and roots [19,20]. The discrepancy in the muscle and function may explain such differences. The thickness of the cortical bone layer may also be affected by muscle activity [21]. 

This study compares pre-treatment (T0) and post-treatment (T1) with LLLT concerning the IRW. At L4, measured IRW values of #23–#24 were significantly different (*p* = 0.039). At T0 and T1, the measured values were 3.893 ± 0.127 and 4.104 ± 0.334 (mean ± SD), respectively. Purmal et al. (2013) [8], Poggio et al. (2006) [15], and Bittencourt et al. (2011) [16] found measured values of 4.07 ± 0.32, 4.3, and 2.7 at T0, respectively. However, the after-effects of the current study could not be compared because no other study has been published looking at the bony changes in IRW association with LLLT vs. non-LLLT using pre- and post-treatment CBCT of FOT cases. Noteworthy, differences in genetic makeup may explain the different IRW outcomes [22,23].

This research evaluated the effects of LLLT on IRW bony changes after FOT using 3DCBCT. The results introduce a novel noninvasive technique to achieve a better orthodontic treatment process than the conventional treatment. The outcomes of IRW in two different groups at all four levels were generally insignificant. The outcome of the study illustrates the effects of LLLT on IRW bony changes using 3DCBCT. Therefore, this study explored IRW bony changes in two different treatment modality groups, LLLT and non-LLLT, using 3DCBCT subsequently after OTM in FOT cases. In addition, this study supports the efficiency of the technique when using LLLT during regular orthodontic visits. Hence, practitioners can offer this new technique to their patients. Although the differences in IRW bony changes are mostly insignificant, LLLT has favorable effects in relation to orthodontic pain perception [4,5], OTM [4], and root resorption [6].

Knowing the extent of orthodontic malocclusion in patients after FOT, IRW before starting any FOT, and being able to discuss such information with the patient, parents, and guardians allows choosing the most suitable FOT in relation to root position and parallelism. Proper root position is necessary for successful orthodontic treatment that is stable, functional, and esthetic. Typically, the primary focus during orthodontic treatment is on crown position rather than root position because roots are not clinically visible and generally not directly involved with esthetics and occlusion [24,25,26]. Root position plays a role in periodontal health, restorative treatment, and occlusal function [26,27,28,29]. Radiographs often reveal crown alignment errors in teeth with poor root angulation. Furthermore, the American Board of Orthodontics (ABO) recommends assessing root parallelism and deducts points if the roots of adjacent teeth are not parallel with each other or if they come in contact with each other [30]. The ABO recommends the use of panoramic radiographs to monitor root alignment even though previous reports and the ABO have acknowledged that panoramic radiographs do not accurately depict root position [31,32].

Limitations: This study pioneered applying LLLT before FOT to later measure the effects on IRW bony changes using 3DCBCT with negative data in a limited environment. Even though the recommended sample size was used, considering the limitations of the current study, replication of the same protocol with a larger sample may give different results. Furthermore, this study was conducted in one center, so conducting the study in more than one center may result in different outcomes. There may be significant differences depending on the setting of LLLT, sex, and age. Finally, a future study measuring the long-term IRW bony changes in the retention phase and after relapse is recommended and may give different insights.

## 5. Conclusions

The outcomes of IRW bony changes as seen in 3DCBCT images of orthodontic patients after FOT with LLLT and without LLLT revealed insignificant differences. We sought to determine the usefulness, or lack thereof, of a treatment intervention taking into account only one variable, the amount of IRW bony changes. Further investigation is needed into other variables and at different centers to confirm or refute our conclusions. Given the outcomes of this study, mostly insignificant differences in IRW bony changes were observed with CBCT before and after treatment with LLLT in patients who underwent FOT.

## Figures and Tables

**Table 1:** IRW of the maxilla (both sides) at various levels. LLLT and non-LLLT differences between pre- and post-treatment groups—middle quadrant teeth.

Variable	Level	LLLT vs. Non-LLLT	T0			T1			T0 vs. T1	T0 vs. T1
			Mean	SD	*p* Value	Mean	SD	*p* Value	*p* Value	*p* Value
#11–#21	L1	LLLT	4.015	0.644	0.708	3.938	0.385	1.000	0.685	
		Non-LLLT	3.944	0.379	0.709	3.938	0.385	1.000		0.966
	L2	LLLT	4.318	0.605	0.924	4.271	0.484	1.000	0.830	
		Non-LLLT	4.300	0.442	0.924	4.271	0.484	1.000		0.875
	L3	LLLT	4.446	0.500	0.346	4.613	0.491	1.000	0.425	
		Non-LLLT	4.614	0.490	0.346	4.613	0.491	1.000		0.994
	L4	LLLT	4.514	0.707	0.628	4.666	0.734	1.000	0.567	
		Non-LLLT	4.639	0.743	0.628	4.666	0.734	1.000		0.915

**Table 2:** IRW of the maxilla (both sides) at various levels. LLLT and non-LLLT differences among pre- and post-treatment groups—anterior quadrant teeth.

Variable	Level	LLLT vs. Non-LLLT	T0			T1			T0 vs. T1	T0 vs. T1
			Mean	SD	*p* Value	Mean	SD	*p* Value	*p* Value	*p* Value
#13–#12	L1	LLLT	2.093	0.173	0.675	2.184	0.248	1.000	0.241	
		Non-LLLT	2.118	0.156	0.675	2.184	0.248	1.000		0.393
	L2	LLLT	2.554	0.224	0.944	2.587	0.459	1.000	0.793	
		Non-LLLT	2.548	0.227	0.944	2.587	0.459	1.000		0.776
	L3	LLLT	2.795	0.201	0.779	3.028	0.464	1.000	0.096	
		Non-LLLT	2.774	0.216	0.779	3.028	0.464	1.000		0.055
	L4	LLLT	3.484	0.296	0.761	3.624	0.492	1.000	0.409	
		Non-LLLT	3.448	0.365	0.761	3.624	0.492	1.000		0.257
#12–#11	L1	LLLT	2.580	0.390	0.609	2.727	0.441	1.000	0.321	
		Non-LLLT	2.654	0.416	0.609	2.727	0.441	1.000		0.598
	L2	LLLT	2.928	0.386	0.227	3.173	0.432	1.000	0.144	
		Non-LLLT	3.103	0.419	0.227	3.173	0.432	1.000		0.595
	L3	LLLT	3.113	0.540	0.181	3.457	0.587	1.000	0.142	
		Non-LLLT	3.388	0.596	0.181	3.457	0.587	1.000		0.755
	L4	LLLT	3.723	0.522	0.387	3.944	0.483	1.000	0.278	
		Non-LLLT	3.883	0.509	0.387	3.944	0.483	1.000		0.741
#11–#21	L1	LLLT	4.015	0.644	0.708	3.938	0.385	1.000	0.685	
		Non-LLLT	3.944	0.379	0.709	3.938	0.385	1.000		0.966
	L2	LLLT	4.318	0.605	0.924	4.271	0.484	1.000	0.830	
		Non-LLLT	4.300	0.442	0.924	4.271	0.484	1.000		0.875
	L3	LLLT	4.446	0.500	0.346	4.613	0.491	1.000	0.425	
		Non-LLLT	4.614	0.490	0.346	4.613	0.491	1.000		0.994
	L4	LLLT	4.514	0.707	0.628	4.666	0.734	1.000	0.567	
		Non-LLLT	4.639	0.743	0.628	4.666	0.734	1.000		0.915
#21–#22	L1	LLLT	2.154	0.278	0.049	2.417	0.413	1.000	0.093	
		Non-LLLT	2.409	0.413	0.050	2.417	0.413	1.000		0.954
	L2	LLLT	2.627	0.347	0.130	2.856	0.508	1.000	0.209	
		Non-LLLT	2.866	0.505	0.131	2.856	0.508	1.000		0.958
	L3	LLLT	2.908	0.481	0.073	3.218	0.581	1.000	0.203	
		Non-LLLT	3.258	0.580	0.073	3.218	0.581	1.000		0.835
	L4	LLLT	3.674	0.444	0.185	3.854	0.496	1.000	0.387	
		Non-LLLT	3.898	0.487	0.185	3.854	0.496	1.000		0.776
#22–#23	L1	LLLT	2.930	0.162	0.033	2.981	0.350	1.000	0.657	
		Non-LLLT	3.054	0.151	0.033	2.981	0.350	1.000		0.427
	L2	LLLT	3.438	0.225	0.784	3.321	0.414	1.000	0.337	
		Non-LLLT	3.418	0.182	0.784	3.321	0.414	1.000		0.447
	L3	LLLT	3.878	0.191	0.789	3.823	0.427	1.000	0.641	
		Non-LLLT	3.860	0.189	0.789	3.823	0.427	1.000		0.764
	L4	LLLT	4.050	0.195	0.122	4.206	0.447	1.000	0.258	
		Non-LLLT	4.156	0.182	0.122	4.206	0.447	1.000		0.719

**Table 3:** IRW of the maxilla (both sides) at various levels. LLLT and non-LLLT differences among pre- and post-treatment groups—posterior right quadrant teeth.

Variable	Level	LLLT vs. Non-LLLT	T0			T1			T0 vs. T1	T0 vs. T1
			Mean	SD	*p* Value	Mean	SD	*p* Value	*p* Value	*p* Value
#17–#16	L1	LLLT	3.684	0.277	0.372	3.641	0.349	1.000	0.743	
		Non-LLLT	3.584	0.339	0.372	3.641	0.349	1.000		0.656
	L2	LLLT	4.225	0.645	0.103	3.928	0.444	1.000	0.169	
		Non-LLLT	3.899	0.431	0.105	3.928	0.444	1.000		0.870
	L3	LLLT	4.256	0.554	0.408	4.167	0.366	1.000	0.580	
		Non-LLLT	4.119	0.346	0.410	4.167	0.366	1.000		0.739
	L4	LLLT	4.795	0.684	0.173	4.549	0.469	1.000	0.151	
		Non-LLLT	4.506	0.467	0.174	4.549	0.469	1.000		0.825
#16–#15	L1	LLLT	2.918	0.342	0.493	3.022	0.328	1.000	0.416	
		Non-LLLT	2.995	0.288	0.493	3.022	0.328	1.000		0.729
	L2	LLLT	3.554	0.631	0.167	3.324	0.287	1.000	0.162	
		Non-LLLT	3.311	0.270	0.171	3.324	0.287	1.000		0.864
	L3	LLLT	3.763	0.576	0.498	3.649	0.327	1.000	0.498	
		Non-LLLT	3.649	0.327	0.499	3.649	0.327	1.000		1.000
	L4	LLLT	4.052	0.369	0.930	4.078	0.390	1.000	0.854	
		Non-LLLT	4.064	0.384	0.930	4.078	0.390	1.000		0.924
#15–#14	L1	LLLT	2.888	0.379	0.221	3.013	0.315	1.000	0.269	
		Non-LLLT	3.055	0.379	0.221	3.013	0.315	1.000		0.625
	L2	LLLT	3.644	0.651	0.952	3.589	0.491	1.000	0.776	
		Non-LLLT	3.631	0.514	0.952	3.589	0.491	1.000		0.795
	L3	LLLT	3.819	0.420	0.261	3.962	0.410	1.000	0.327	
		Non-LLLT	3.989	0.417	0.261	3.962	0.410	1.000		0.822
	L4	LLLT	4.224	0.513	0.535	4.324	0.534	1.000	0.629	
		Non-LLLT	4.341	0.539	0.535	4.324	0.534	1.000		0.926
#14–#13	L1	LLLT	2.653	0.104	0.265	3.044	0.401	0.972	0.001	
		Non-LLLT	2.595	0.174	0.267	3.039	0.470	0.972		0.002
	L2	LLLT	3.161	0.152	0.814	3.449	0.371	0.973	0.011	
		Non-LLLT	3.144	0.244	0.814	3.454	0.449	0.973		0.034
	L3	LLLT	3.616	0.254	0.780	3.855	0.352	0.862	0.055	
		Non-LLLT	3.588	0.298	0.780	3.881	0.469	0.862		0.061
	L4	LLLT	3.981	0.189	0.783	4.215	0.351	0.813	0.030	
		Non-LLLT	3.959	0.240	0.783	4.248	0.424	0.814		0.041

**Table 4:** IRW of the maxilla (both sides) at various levels. LLLT and non-LLLT differences among pre- and post-treatment groups—posterior left quadrant teeth.

Variable	Level	LLLT vs. Non-LLLT	T0			T1			T0 vs. T1	T0 vs. T1
			Mean	SD	*p* Value	Mean	SD	*p* Value	*p* Value	*p* Value
#23–#24	L1	LLLT	2.962	0.164	0.004	3.143	0.352	0.957	0.108	
		Non-LLLT	3.182	0.230	0.004	3.136	0.364	0.957		0.660
	L2	LLLT	3.631	0.212	0.886	3.509	0.317	0.853	0.233	
		Non-LLLT	3.619	0.275	0.887	3.530	0.307	0.853		0.431
	L3	LLLT	3.628	0.157	0.047	3.726	0.318	0.867	0.324	
		Non-LLLT	3.777	0.239	0.048	3.745	0.310	0.867		0.744
	L4	LLLT	3.893	0.127	0.043	4.104	0.334	0.736	0.039	
		Non-LLLT	4.049	0.266	0.046	4.143	0.319	0.736		0.356
#24–#25	L1	LLLT	2.758	0.234	0.108	2.954	0.385	1.000	0.083	
		Non-LLLT	2.945	0.388	0.110	2.954	0.385	1.000		0.943
	L2	LLLT	3.424	0.423	0.898	3.463	0.421	1.000	0.803	
		Non-LLLT	3.444	0.424	0.898	3.463	0.421	1.000		0.910
	L3	LLLT	3.754	0.281	0.445	3.861	0.397	1.000	0.393	
		Non-LLLT	3.849	0.399	0.446	3.861	0.397	1.000		0.938
	L4	LLLT	4.276	0.395	1.000	4.270	0.434	1.000	0.972	
		Non-LLLT	4.276	0.433	1.000	4.270	0.434	1.000		0.973
#25–#26	L1	LLLT	3.076	0.599	0.552	3.283	0.711	1.000	0.386	
		Non-LLLT	3.208	0.641	0.552	3.283	0.711	1.000		0.720
	L2	LLLT	3.486	0.601	0.644	3.689	0.716	1.000	0.425	
		Non-LLLT	3.584	0.589	0.644	3.689	0.716	1.000		0.638
	L3	LLLT	3.661	0.417	0.367	3.817	0.510	1.000	0.406	
		Non-LLLT	3.811	0.505	0.367	3.817	0.510	1.000		0.969
	L4	LLLT	4.225	0.402	0.576	4.296	0.518	1.000	0.617	
		Non-LLLT	4.318	0.522	0.576	4.296	0.518	1.000		0.883
#26–#27	L1	LLLT	2.508	0.388	0.210	2.760	0.500	1.000	0.210	
		Non-LLLT	2.706	0.482	0.210	2.760	0.500	1.000		0.741
	L2	LLLT	3.561	0.808	0.108	3.224	0.424	1.000	0.120	
		Non-LLLT	3.183	0.423	0.112	3.224	0.424	1.000		0.793
	L3	LLLT	3.694	0.708	0.338	3.592	0.498	1.000	0.641	
		Non-LLLT	3.489	0.457	0.340	3.592	0.498	1.000		0.571
	L4	LLLT	3.921	0.505	0.588	4.131	0.459	1.000	0.210	
		Non-LLLT	4.014	0.463	0.589	4.131	0.459	1.000		0.510

## Data Availability

The data used to support the findings of this study are included in the article.

## References

[1] Ackerman M.B. (2007). Enhancement Orthodontics: Theory and Practice.

[2] Fink D.F., Smith R.J. (1992). The duration of orthodontic treatment. Am. J. Orthod. Dentofac. Orthop..

[3] Jawad M.M., Husein A., Alam M.K., Hassan R., Shaari R. (2012). Overview of non-invasive factors (low level laser and low intensity pulsed ultrasound) accelerating tooth movement during orthodontic treatment. Lasers Med. Sci..

[4] Qamruddin I., Alam M.K., Mahroof V., Fida M., Khamis M.F., Husein A. (2017). Effects of low-level laser irradiation on the rate of orthodontic tooth movement and associated pain with self-ligating brackets. Am. J. Orthod. Dentofac. Orthop..

[5] Alam M.K. (2019). Laser-Assisted Orthodontic Tooth Movement in Saudi Population: A Prospective Clinical Intervention of Low-Level Laser Therapy in the 1st Week of Pain Perception in Four Treatment Modalities. Pain Res. Manag..

[6] Alam M.K., Ganji K.K., Alfawzan A.A., Manay S.M., Srivastava K.C., Chaudhari P.K., Hosni H.A., Alswairki H.J., Alansari R.A. (2022). Ectopic Eye Tooth Management: Photobiomodulation/Low-Level Laser Emission Role in Root Resorption after Fixed Orthodontic Treatment. Healthcare.

[7] Patel S., Dawood A., Whaites E., Ford T.P. (2009). New dimensions in endodontic imaging: Part 1. Conventional and alternative radiographic systems. Int. Endod. J..

[8] Purmal K., Alam M.K., Pohchi A., Razak N.H.A. (2013). 3D Mapping of Safe and Danger Zones in the Maxilla and Mandible for the Placement of Intermaxillary Fixation Screws. PLoS ONE.

[9] Lee K.-J., Joo E., Kim K.-D., Lee J.-S., Park Y.-C., Yu H.-S. (2009). Computed tomographic analysis of tooth-bearing alveolar bone for orthodontic miniscrew placement. Am. J. Orthod. Dentofac. Orthop..

[10] Nalcaci R., Cokakoglu S. (2013). Lasers in Orthodontics. Eur. J. Dent..

[11] Mah J.K., Huang J.C., Choo H. (2010). Practical applications of conebeam computed tomography in orthodontics. J. Am. Dent. Assoc..

[12] American Academy of Oral and Maxillofacial Radiology (2013). Clinical recommendations regarding use of cone beam computed tomography in orthodontics. Position statement by the American Academy of Oral and Maxillofacial Radiology. Oral Surg. Oral Med. Oral Pathol. Oral Radio. Endod..

[13] Alqerban A., Jacobs R., Souza P.C., Willems G. (2009). In-vitro comparison of 2 cone-beam computed tomography systems and panoramic imaging for detecting simulated canine impaction-induced external root resorption in maxillary lateral incisors. Am. J. Orthod. Dentofacial Orthop..

[14] Sherrard J.F., Rossouw P.E., Benson B.W., Carrillo R., Buschang P.H. (2010). Accuracy and reliability of tooth and root lengths measured on cone-beam computed tomographs. Am. J. Orthod. Dentofac. Orthop..

[15] Poggio P.M., Incorvati C., Velo S., Carano A. (2006). “Safe zones”: A guide for miniscrew positioning in the maxillary and man-dibular arch. Angle Orthod..

[16] Bittencourt L.P., Raymundo M.V., Mucha J.N. (2011). The optimal position for insertion of orthodontic miniscrews. RevistaOdonto-ciencia.

[17] Malmgren O., Goldson L., Hill C., Orwin A., Petrini L., Lundberg M. (1982). Root resorption after orthodontic treatment of traumatized teeth. Am. J. Orthod..

[18] Lavelle C.L. (1972). Maxillary and mandibular tooth size in different racial groups adn in different occlusal categories. Am. J. Orthod..

[19] Bishara S.E., Jakobsen J.R., Abdallah E.M., Garcia A.F. (1989). Comparisons of mesiodistal and buccolingual crown dimensions of the permanent teeth in three populations from Egypt, Mexico and the United States. Am. J. Orthod. Dentofacial Orthop..

[20] Harris E.F., Rathbun T.A. (1991). Ethnic differences in the apportionment of tooth sizes. Adv. Dent. Anthropol..

[21] Thongudomporn U., Chongsuvivatwong V., Geater A.F. (2009). The effect of maximum bite force on alveolar bone morphology. Orthod. Craniofac. Res..

[22] Janson G.R., Canto G.D.L., Martins D.R., Henriques J.F.C., de Freitas M.R. (2000). A radiographic comparison of apical root resorption after orthodontic treatment with 3 different fixed appliance techniques. Am. J. Orthod. Dentofac. Orthop..

[23] Jiang R.-P., McDonald J.P., Fu M.-K. (2010). Root resorption before and after orthodontic treatment: A clinical study of contributory factors. Eur. J. Orthod..

[24] Germane N., Bentley B.E., Isaacson R.J. (1989). Three biologic variables modifying faciolingual tooth angulation by straight-wire appliances. Am. J. Orthod. Dentofac. Orthop..

[25] Bryant R., Sadowsky P., Dent M., Hazelrig J. (1984). Variability in three morphologic features of the permanent maxillary central incisor. Am. J. Orthod..

[26] Dewel B. (1949). Clinical observations on the axial inclination of teeth. Am. J. Orthod..

[27] Vermylen K., De Quincey G.N.T., Hof M.A.V., Wolffe G.N., Renggli H.H. (2005). Classification, reproducibility and prevalence of root proximity in periodontal patients. J. Clin. Periodontol..

[28] Vermylen K., De Quincey G.N.T., Wolffe G.N., Hof M.A.V., Renggli H.H. (2005). Root proximity as a risk marker for periodontal disease: A case-control study. J. Clin. Periodontol..

[29] Klassman B., Zucker H.W. (1969). Treatment of a Periodontal Defect Resulting from Improper Tooth Alignment and Local Factors. J. Periodontol..

[30] Casko J.S., Vaden J.L., Kokich V.G., Damone J., James R.D., Cangialosi T.J., Riolo M.L., Owens S.E., Bills E.D. (1998). Objective grading system for dental casts and panoramic radiographs. Am. J. Orthod. Dentofac. Orthop..

[31] Garcia-Figueroa M.A., Raboud D.W., Lam E.W., Heo G., Major P.W. (2008). Effect of buccolingual root angulation on the mesiodistal angulation shown on panoramic radiographs. Am. J. Orthod. Dentofac. Orthop..

[32] Owens A.M., Johal A. (2008). Near-End of Treatment Panoramic Radiograph in the Assessment of Mesiodistal Root Angulation. Angle Orthod..

